# The Genetics of Resistance to *Morinda* Fruit Toxin During the Postembryonic Stages in *Drosophila sechellia*

**DOI:** 10.1534/g3.114.015073

**Published:** 2015-07-29

**Authors:** Yan Huang, Deniz Erezyilmaz

**Affiliations:** Department of Biochemistry and Cell Biology and Center for Developmental Genetics, Stony Brook University, Stony Brook, New York 11794

**Keywords:** *Drosophila sechellia*, adaptation, ecologic specialization, resistance

## Abstract

Although a great deal has been learned regarding the genetic changes that give rise to adaptation in bacteria and yeast, an understanding of how new complex traits arise in multicellular organisms is far less complete. Many phytophagous insect species are ecological specialists that have adapted to utilize a single host plant. *Drosophila sechellia* is a specialist that utilizes the ripe fruit of *Morinda citrifolia*, which is toxic to its sibling species, *D. simulans*. Here we apply multiplexed shotgun genotyping and QTL analysis to examine the genetic basis of resistance to *M. citrifolia* fruit toxin in interspecific hybrids. We identify a locus of large effect on the third chromosome (QTL-III*_sim_*a) in the *D. simulans* backcross that was not detected in previous analyses. We also identify a highly significant QTL of large effect on the X chromosome, QTL-X*_sim_*. Additional smaller-effect loci were also identified in the *D. simulans* and *D. sechellia* backcrosses. We did not detect significant epistasis between loci. Instead, our analysis reveals large and smaller-effect loci that contribute to *M. citrifolia* resistance additively. The additive effect of each locus suggests that partial resistance to lower levels of *M. citrifolia* toxin could be passed through introgression from *D. sechellia* to *D. simulans* in nature. The identification of the major effect loci, QTL-III*_sim_*a and QTL-X*_sim_*, is an important step toward identifying the molecular basis of adaptation in a multicellular organism.

How genetic architecture may contribute to the emergence of ecological specialization remains an open question (Forister *et al.* 2012). Genetic mapping methods between recently diverged species have made it possible to assess the relative contributions of dominance, epistasis, complexity, and effect size on the emergence of adaptive traits. Studies of adaptation in bacteria have revealed that adaptive change occurs in discreet jumps through large-effect mutations. But bacteria have a high mutation rate and rapid rate of reproduction, and it is not known if adaptation in complex, multicellular organisms also occurs through changes of large effect (Chouard 2010).

The fruit fly, *Drosophila sechellia*, provides a clear example of adaptive specialization within the *Drosophila simulans* clade, which includes *D. simulans*, *D. sechellia*, and *D. mauritiana*. The three species emerged on islands in the Indian Ocean. *D. simulans* probably evolved in Madagascar, but *D. sechellia* and *D. mauritiana* are endemic to the Seychelles and Mascarene Islands, respectively (Lachaise *et al.* 1986). Molecular analysis suggests that *D. sechellia* diverged from its siblings ∼413,000 years ago and *D. mauritiana* diverged ∼263,000 years ago (Kliman *et al.* 2000), although a genome-wide analysis indicates a nearly simultaneous split ∼242,000 years ago, with subsequent admixture since speciation (Garrigan *et al.* 2012). Although both *D. simulans* and *D. mauritiana* exploit a wide variety of fruit, *D. sechellia* is a specialist, and wild *D. sechellia* are found, most frequently, on the fruit of *Morinda citrifolia* (Tsacas and Bachli 1981; Louis and David 1986; Matute and Ayroles 2014), which is toxic to other species of *Drosophila* when it is ripe (Louis and David 1986; R’Kha *et al.* 1991; Legal *et al.* 1994). Adult *D. sechellia* are attracted to ripe *M. citrifolia*, and oviposition behavior is stimulated by *M. citrifolia* fruit volatiles (R’Kha *et al.* 1991; Legal *et al.* 1992; Higa and Fuyama. 1993; Legal *et al.* 1999). Neither unripe nor fermenting *M. citrifolia* fruit is toxic to *D. simulans* or *D. melanogaster* (Legal *et al.* 1992; Farine *et al.* 1996; Legal *et al.* 1994). Gas chromatography–mass spectrometry analysis of ripe *M. citrifolia* fruit revealed an abundance of the compound octanoic acid, a linear eight-chain fatty acid that was prevalent in ripe fruit, but not unripe or fermenting fruit (Legal *et al.* 1994; Farine *et al.* 1996; Legal *et al.* 1999). Octanoic acid is toxic to *D. simulans*, *D. mauritiana*, and *D. melanogaster* at all stages of development (R’Kha *et al.* 1991; Legal *et al.* 1992; Jones 1998, 2001).

The genetic basis for octanoic acid resistance varies with developmental stage. Using 15 genetic markers, Jones (1998) found that resistance in adults is conveyed by a region on 3R, two regions on the X, and additional factors on the second chromosome that could not be localized. To identify larval resistance genes, Jones (2001) used 11 genetic markers and found a major effect locus on the right arm of the third chromosome in the *D. simulans* backcross, with smaller-effect loci on 2L and 2R, as well as sex-specific epistatic interactions. A scan for loci in progeny from the *D. sechellia* backcross revealed a single locus of large effect on 3R and a small-effect interaction between 2L and 2R. Although these analyses have established that octanoic acid resistance in *D. sechellia* is a trait of moderate complexity, the low resolution of these pregenome studies makes it difficult to guess the number or location of loci that are involved. In addition, mapping at lower resolution increases the likelihood of finding "ghost QTL," which are QTL peaks created between two true loci when intervening markers are not available to resolve them (Broman and Speed 1999).

With the emergence of low-cost next-generation sequencing, it has become possible to assign ancestry at each polymorphic site that exists between two genomes. DNA barcoding methods make high-density genotyping cost-effective by multiplexing many individual genomes into a single sequencing library (Baird *et al.* 2008; Andolfatto *et al.* 2011). To create a high-resolution view of the genetics of *M. citrifolia* resistance in *D. sechellia*, we have used multiplexed shotgun genotyping (MSG) to genotype *M. citrifolia*–resistant and *M. citrifolia*–sensitive larvae at hundreds of thousands of markers. Here we show that the genetic basis for larval resistance in *D. sechellia*, from the second larval instar (L2) to puparium formation, is conferred through two large-effect loci in combination with multiple smaller-effect loci.

## Materials and Methods

### Larval resistance assays

To generate synchronized larvae, we transferred individual late first instar (L1) larvae from timed egg lays to separate media dishes. After 2 hr, L2 larvae were identified and then moved to *M. citrifolia*, the octanoic acid test medium, or to regular fly food. The midpoint of the 2-hr interval is taken as the time of the L1–L2 molt. Molting larvae were identified by the presence of double mouth hooks, posterior to anterior peristalsis movements or ecdysis.

To generate the octanoic acid test medium, octanoic acid from Sigma (St. Louis, MO) was diluted in water and mixed with 0.25 g nutritional yeast and 2.0 g Carolina instant *Drosophila* food, Formula 4-24 (Burlington, NC), and used within 1 hr. For the octanoic acid dose-response tests, larvae were staged at the L1 to L2 molt and moved to octanoic acid medium 3 hr after the onset of the second instar to avoid handling during the L2 molt. The number of pupae was recorded after 3 d. Larvae were tested in groups of 30.

*M. citrifolia* was grown in Hawaii and shipped frozen overnight to New York (a generous gift from Prof. Scot Nelson, University of Hawaii). We find that each fruit from this source always produces 100% lethality in *D. simulans*. For the *M. citrifolia* resistance assays used to phenotype larvae for QTL mapping, L2 larvae were individually selected from 6-hr to 12-hr collections and transferred to thawed *M. citrifolia* fruit that had been frozen at the "translucent gray" stage while still firm (Chan-Blanco *et al.* 2006). Frozen fruits were thawed overnight at room temperature and used within 24 hr. The larvae on *M. citrifolia* were monitored for the next 6 hr. Those larvae that stopped moving (but were not undergoing peristalsis movements of a molt) were considered *M. citrifolia*–sensitive. Those that survived to form puparia were considered resistant.

### Genotyping

We generated recombinant mapping populations by crossing female *D. simulans^Nueva^* to male *D. sechellia^w30^*. The virgin F1 hybrid females were backcrossed to either parental stock.

For multiplexed shotgun genotyping, DNA was extracted from individual flies using a modified version of the Purgene protocol (Erezyilmaz and Stern 2013). Custom barcode adapters (Supporting Information, File S1) were ligated onto genomic DNA, and libraries of 384 barcoded individual genomes were processed according to Andolfatto *et al.* (2011). For our *D. simulans* backcross, we sequenced two libraries of 384 barcoded genomic DNA samples from resistant pupae and two libraries with 384 individual sensitive larvae each. Each library of 384 barcoded individuals was sequenced on an Illumina HiSeq (SRS645327). For the *D. sechellia^w30^* backcross, we combined barcoded DNA from 186 resistant larvae with 192 sensitive larvae into a separate lane (SRS645326). The reads of all our libraries were mapped to *D. simulans* release 1.3 that was updated with the parental strains *D. simulans^Nueva^* (SRS643607) and *D. sechellia^w30^* (ID: 2867884) with the UpdateParental function of the MSG package (Github:JaneliaSciComp/msg). We use the *D. simulans* r1.3 throughout the article except for Table 6, in which the locations of QTL are converted into coordinates in the current genome assembly according to chromosome arm (based on Hu *et al.* 2012). The MSG package was installed on Galaxy Cloudman on the Amazon Elastic Compute Cloud. Configuration files, msg.cfg, and update.cfg with selected parameters are available as Supporting Information, File S2 and File S3.

We identified a species-specific indel near 22.36 from the locations reported in Erezyilmaz and Stern (2013). The primers 3R22.36f: 5′ CAGTACACAATGGTGGGCAT 3′ and 3R22.36r: 5′TTTGGTCCAAAAGGAAGCTGA 3′ straddle a region of a *D. simulans*–*D. sechellia* alignment that contains a 20-bp deletion in *D. simulans*. The PCR-amplified products were visualized on a gel made with 2.5% Lonza MetaPhor agarose (Basel, Switzerland).

### Post-MSG processing

The MSG program creates far more markers than the number of recombination events in our cross and most genotype data are redundant. We therefore used the custom python script, pull_thin.py, created by David Stern (Janelia Farm and HHMI; Supporting Information) to delete genotype data for markers that do not straddle at least one recombination event in any individual (Cande *et al.* 2012). The *D. sechellia^w30^* backcross data set was thinned from 406,475 markers to 8206 markers. The *D. simulans* backcross dataset was thinned from 618,493 markers to 12,461 markers.

### QTL mapping

We treated *M. citrifolia* resistance as a binary trait and scored survivorship to puparium formation. We used the *detectable* and *powercalc* functions of the R package, R/QTL Design (Sen *et al.* 2007), to determine the power of our QTL experiments to detect given effect sizes. We used the *scanone* function in R/qtl (Broman *et al.* 2003) to scan for QTL and plotted the results in R. For composite interval mapping, we used the *cim* function of R/qtl. To assess the statistical significance of our LOD scores in the one-dimensional analyses, we performed permutation analysis with 1000 replicates. For two-dimensional scans, we used reduced datasets that contained 190 markers for the *D. sechellia^w30^* backcross and 166 markers for the *D. simulans* backcross. We analyzed the reduced datasets using the *scantwo* function in R/qtl and in the R/qtl interface, J/qtl (Smith *et al.* 2009). The statistical significance of our two-dimensional scans was assessed using 500 permutation replicates. The fits of the models were tested using the *fitqtl* function of R/qtl (Broman *et al.* 2003).

We determined the sex of larvae and pupae through genotyping the X chromosome. When the X chromosome was nonrecombinant and derived from the same parent as the backcross direction, however, we were unable to determine the sex of the individual fly. For tests of sex as a covariate we removed flies with ambiguous sex, approximately 10% of all individuals, and performed scans with a smaller dataset.

### Fly strains

The QTL experiments were performed with a white-eyed mutant of the strain *D. simulans^Nueva^* (San Diego Stock Center #14021-0251.006) and *D. sechellia^w30^* (#14021-0248.30). The mutant strains *D. simulans^cutsy,ro,ca^* (San Diego Stock Center #14021-0251.116) and *D. simulans^jv,st,e,p^* (San Diego Stock Center #14021-0251.174) were used in marker association experiments. We used an inbred line of *D. sechellia* (San Diego Stock Center #14021-0248.13), *D. sechellia^D1A1C^*, for the marker association experiments with *D. simulans^cutsy,ro,ca^*. Crosses between *D. sechellia^D1A1C^* and an inbred strain of *D. simulans* from Madagascar (Tsimbazaza),were used for reciprocal backcrosses to test the effect of the X chromosome.

### Data availability

All fly strains are available from the San Diego Stock Center. Sequence Data are available on NIH SRA as Bioproject PRJNA253193.

## Results

### Resistance during the late larval stages and puparium formation

**Figure 1:**
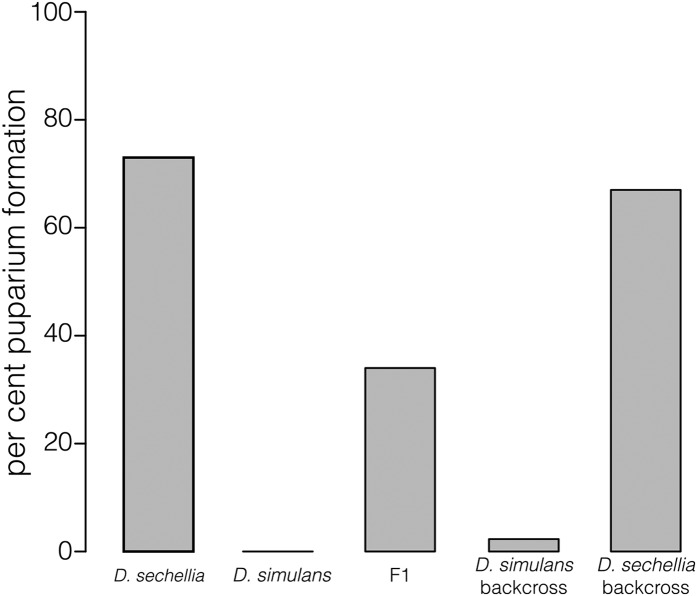
Survival to puparium formation of larvae transferred to ripe *M. citrifolia* fruit. F1 = *D. simulans*^Nueva^/*D. sechellia^w30^* hybrids. *D. simulans* backcross = progeny from F1 females and *D. simulans*^Nueva^ males. *D. sechellia* backcross = progeny from F1 females and *D. sechellia^w30^* males.

After hatching, *Drosophila* progress through three larval stages (L1–L3). The onset of metamorphosis occurs as the third instar larva becomes immobile and forms a puparium. *D. sechellia* tends to pupariate in their host, *M. citrifolia*, suggesting that the immature stages of this species are spent entirely in the host fruit. Ripening in *M. citrifolia* occurs as hard green fruit turns pale yellow, softens, and becomes a translucent gray color as the fruit ripens (Chan-Blanco *et al.* 2006). We used fruits at the "translucent gray" stage and devised a high-throughput method for selecting juvenile *Drosophila* for resistance. Staged L2 larvae are transferred from fly food to *M. citrifolia* fruit and allowed to feed and form puparia. Under these conditions, 73% of *D. sechellia^w30^* larvae (N= 180), but no *D. simulans^Nueva^* larvae (N = 240), survived to form puparia (Figure 1).

**Figure 2:**
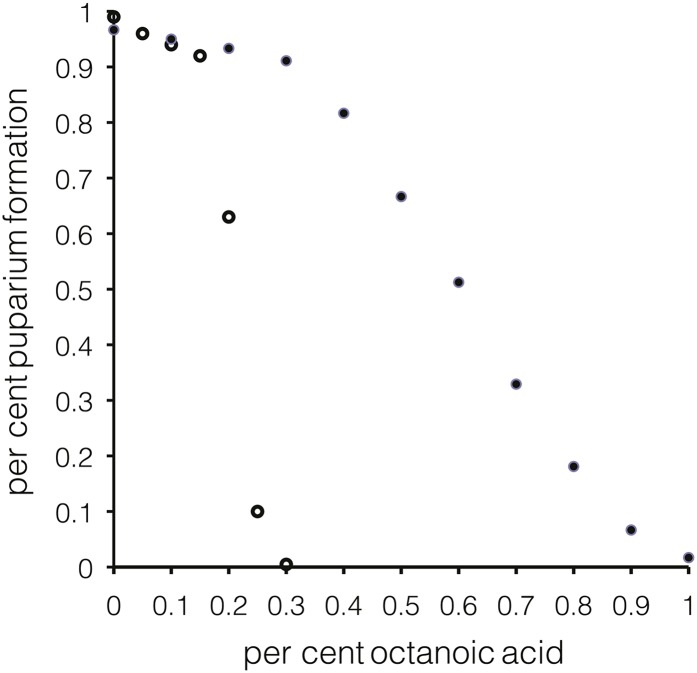
Percentage of larvae that form puparia after transfer to octanoic acid during the second larval instar. The percentage of octanoic acid within *Drosophila* food is given on the x-axis. Filled circles represent 2070 staged *D. sechellia^w30^* larvae that were tested in groups of 30. Between 60 and 240 larvae were used for each data point. Open circles indicate data for 1620 *D. simulans*^Nueva^ larvae. Between 180 and 270 larvae were used for each data point.

To compare the efficacy of our *M. citrifolia* fruit assay to the effects of pure octanoic acid, we examined the dose-response relationship between octanoic acid concentrations in fly media for the stages from L2 to pupariation. *D. sechellia^w30^* had approximately three-fold greater resistance to octanoic acid than *D. simulans^Nueva^* (Figure 2; *D. sechellia^w30^* LD_50_ = ∼0.6%; *D. simulans^Nueva^* LD_50_ = ∼0.22% octanoic acid). The level of toxicity at which 73% of *D. sechellia^w30^* larvae died approximately corresponds to 0.46% octanoic acid.

**Figure 3:**
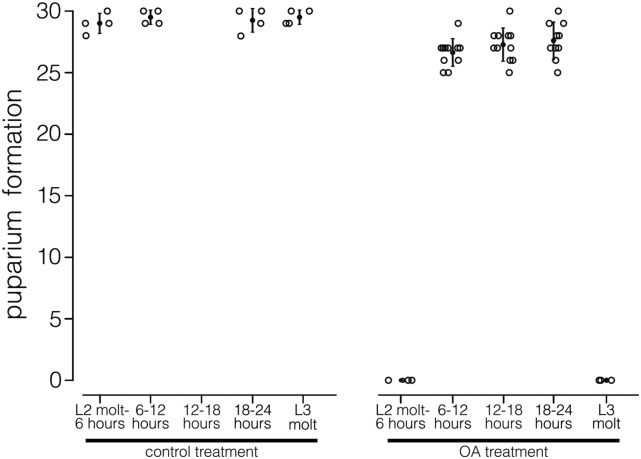
Survivorship to puparium formation after 6-hr treatments during the second larval instar for control (left panel) and 0.2% octanoic acid (OA) treatments. Each open circle represents a test group of 30 larvae. Filled circle indicates the mean and the error bars show SEs.

Most L2 larvae did not die on contact with octanoic acid. Lethality, instead, occurred during a range of time after transfer to octanoic acid–containing medium. We next tested the possibility that toxicity in *D. simulans* larvae is due to octanoic acid during specific developmental intervals. We exposed *D. simulans^Nueva^* stage L2 larvae to an intermediate dose, 0.2% of octanoic acid for 6-hr periods, and then returned larvae to octanoic acid–free medium and measured survivorship to the pupal stage (Figure 3). We found that the survivorship of *D. simulans^Nueva^* larvae during the intermolt period (hours 6–24) was comparable to the survivorship of control larvae that were similarly handled but not exposed to octanoic acid (Figure 3). In contrast, all *D. simulans^Nueva^* larvae that were exposed to octanoic acid during either the L1 to L2 molt (N = 90) or the L2 to L3 molt (N = 90) did not survive to form puparia (Figure 3). These data show that at the concentration that we describe here, octanoic acid toxicity during the second larval instar occurs at molts.

### QTL analysis

*D. sechellia* and *D. simulans* produce fertile female offspring when crossed (Lachaise *et al.* 1986). Previous work has shown that resistance to octanoic acid in *D. sechellia* is neither genetically simple nor very complex (R’Kha *et al.* 1991; Jones 2001). We tested the viability of L2 larvae in pieces of *M. citrifolia* fruit and found that overall survivorship of F1 *D. sechellia^w30^*/*D. simulans^Nueva^* hybrids to *M. citrifolia* toxin is intermediate between the survivorship of the two parents (Figure 1). To map resistance loci at high resolution, we screened L2 larvae from backcrosses to either parent in our *M. citrifolia* assays and genotyped individual larvae using MSG (Andolfatto *et al.* 2011).

**Figure 4:**
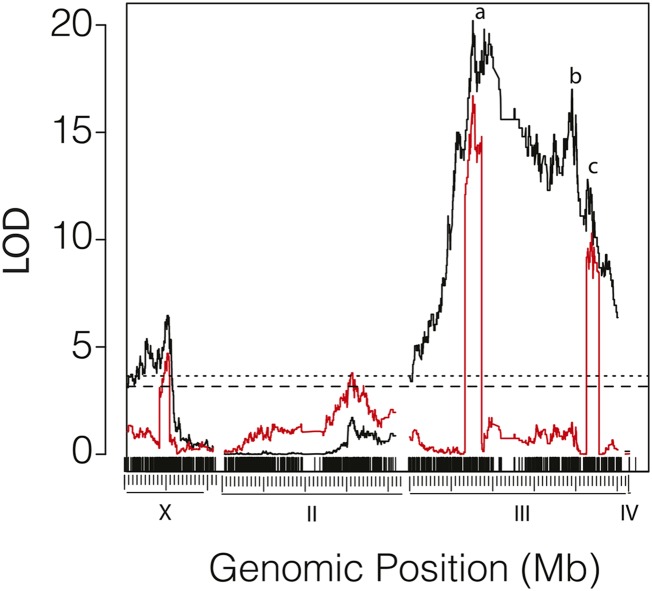
QTL map of *M. citrifolia* resistance in the *D. sechellia^w30^* backcross using interval mapping (black) and composite interval mapping (red). The log of the likelihood ratio (y-axis) is plotted against physical distances (x-axis). The locations of markers are shown in the upper row of vertical ticks. The genomic locations are given in the bottom row of vertical ticks; each large tick is 10 Mbp, and the smaller ticks show 1-Mbp intervals. Dashed and dotted lines indicates the threshold for LOD scores at the 0.05 and 0.01 significance levels, respectively. The locations of QTL-III*_sec_*a (A), QTL-III*_sec_*b (B), and QTL-III*_sec_*c (C) are indicated.

We first examined the resistance of larvae in *M. citrifolia* fruit in 359 individuals from an F1 backcross to *D. sechellia^w30^*. The QTL map created by interval mapping is dominated by QTL on the third chromosome; all regions of the third chromosome are significant at the 99% level (Figure 4). The highest peak is on the left arm of chromosome III at ∼15,860,000 (LOD = 20.2). Inclusion of a neighboring peak at ∼3:18,900,000 did not increase the likelihood of our model significantly (data not shown). We therefore treat this region as one locus, QTL-III*_sec_*a. A second large-effect locus appears on the right arm at ∼3:40,070,000 with LOD of 17.0 (QTL-III*_sec_*b; Figure 4). In addition, two QTL scans indicate that a resistance locus exists on chromosome III at ∼45,180,000 (QTL-III*_sec_*c, LOD = 10.9; Figure 4, Supporting Information, Figure S1), and inclusion of this region improves the fit or our model (Table 1; Table S1). We also found a significant contribution from the X chromosome; the left half is significant at the 95% confidence level, and a peak lies at ∼X:10,680,000 (QTL-X*_sec_*, LOD = 6.5; Figure 4). Loci on the second and the fourth chromosomes did not contribute significantly to resistance. We next analyzed our cross data with composite interval mapping, which uses markers as covariates to eliminate residual variation. Our QTL on the third chromosome refined the significant regions to three QTL: QTL-Xsec, QTL-III*_sec_*a, and QTL-III*_sec_*c (Figure 4, red lines; Figure S3). The best-fit model contains QTL-III*_sec_*a, QTL-III*_sec_*c, and QTL-X*_sec_* interacting additively (Model I, Table 1), although inclusion of QTL-II*_sec_*b could improve the fit of our model modestly (Model II, Table 1). We did not detect significant epistasis between loci in our two-QTL scans (Figure S1).

**Table 1:** Favored QTL models for the *D. simulans* and *D. sechellia* backcrosses

Cross	No. of QTL in Model	LOD of Model	Percent of Variance Explained	QTL Locations	Species Difference %	LOD Drop One*a*
*D. sechellia*	3	31	45.2	X ∼10.8 Mbp	5.9	4.9
Backcross				3 ∼15.9 Mbp	12.3	9.7
Model I				3 ∼40.0 Mbp	9.2	7.3
*D. sechellia*	4	33.8	48.2	X ∼10.8 Mbp	5.7	5.0
Backcross				3 ∼15.9 Mbp	13.4	11.0
Model II				3 ∼40.0 Mbp	2.0	1.7
				3 ∼45.2 Mbp	3.4	2.9
*D. simulans*	5	96.2	38.9	X ∼14.8 Mbp	6.6	18.9
Backcross				2 ∼9.1 Mbp	2.1	6.2
				2 ∼40.8 Mbp	2.1	6.2
				3 ∼5.3 Mbp	2.3	6.5
				3 ∼46.9 Mbp	20.6	54.7

a Log likelihood ratios comparing the full model to a model with the specified QTL removed.

We generated modified datasets to test the effect of sex, because the sex of ∼10% of the individuals in our full scan could not be determined. We found a significant interaction between a marker at 3:15,342,299 and sex; inclusion of this interaction could increase the fit of our model by 2.4 LOD and capture an additional 2.4% of the variation (data not shown).

Given the genetic and environmental variances of our lines, and the number of backcross individuals tested, our *D. sechellia^w30^* backcross QTL experiment is designed to detect effect sizes of 0.19 and 0.175 with 90% and 80% power, respectively. The effect sizes of QTL-III*_sec_*a, QTL-III*_sec_*b, QTL-III*_sec_*c, and QTL-X*_sec_* exceed the threshold for detection with 90% power (Figure S2). Our best-fit models explain 45.2% or 48.2% of the variance in phenotype (Table 1).

**Figure 5:**
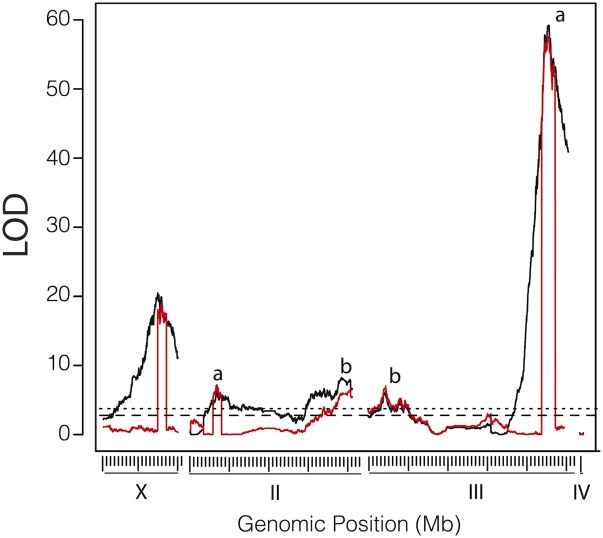
QTL map of *M. citrifolia* resistance in the *D. simulans*^Nueva^ backcross using interval mapping (black) and composite interval mapping (red). (A) LOD scores *vs.* genomic position for the four chromosomes. The upper rug of ticks shows the location of markers, whereas the lower tick marks indicate 1- and 10-Mbp intervals along each chromosome. The significance thresholds for the 0.05 and 0.01 levels of the LOD scores are shown by the dashed and dotted lines, respectively. The lower case letters indicate the locations of QTL-III*_sech_*a, QTL-III*_sech_*b, and QTL-III*_sech_*c. QTL-II*_sech_*a and QTL-II*_sech_*b. (B) Effect plot for QTL-III*_sim_*a showing the phenotypic effect of substituting a *D. simulans*^Nueva^ allele (*sim*) for a *D. sechellia^w30^* allele (*sec*) at the marker 3:46,954,562, where 0 = lethality and 1 = survivorship to puparium formation.

We next examined the resistance of *D. sechellia* to *M. citrifolia* toxin in 1374 progeny of the F1 backcross to *D. simulans^Nueva^*. QTL analysis showed that resistance to *M. citrifolia* toxin is dominated by a QTL of large effect on the right arm of chromosome III at ∼3: 46,880,000 bp (QTL-III*_sim_*a; LOD = 59.2; Figure 5, A and B) and a broad region on the X chromosome between ∼X:10,000,000 and ∼X:18,200,000 that has a peak at ∼X:14,774,000 bp (QTL-X*_sim_*_,_ LOD = 20.1; Figure 5). Larvae with one copy of the *D. sechellia^w30^* allele at QTL-III*_sim_*a were more than twice as likely to survive to pupariate (Figure 5B; Figure S2). We also detected two significant peaks on the left arm of chromosome 3 at ∼3:5,375,000 bp (LOD = 6.13; Figure 5) and at ∼3:9,266,000 bp (LOD = 4.88; Figure 5), although a model including both markers did not significantly increase the likelihood score over a model that includes just one of the two 3L markers (data not shown). We therefore treat these terms as a single locus, QTL-III*_sim_*b. Chromosome 2 contains a significant region on the left arm that peaks at ∼2:9,083,000 (QTL-II*_sim_*a, LOD = 6.3; Figure 5) and a region of significance on the right arm that peaks at ∼2:40,813,000 bp (QTL-II*_sim_*b, LOD = 8.24; Figure 5). Analysis of our cross data with composite interval mapping narrowed the breadth of peaks QTL-X*_sim_*, QTL-II*_sim_*a, and QTL-III*_sim_*a (Figure 5, red lines). We did not detect significant epistasis between QTL in two-dimensional two-QTL scans (Figure S1). Instead, we find that a model that fits our data best consists of QTL-II*_sim_*a QTL-II*_sim_*b, QTL-III*_sim_*a, QTL-III*_sim_*b, and QTL-X*_sim_* interacting additively (Table 1). Inclusion of additional QTL did not improve the fit of our model significantly (Table S1). Finally, we analyzed the effect of sex as a covariate in a modified dataset comprising only *D. simulans* backcross progeny that could be genotyped for sex with certainty. We found a significant interaction between the marker 3:9,061,304 and sex, and inclusion of this interaction could improve the fit of our five-QTL model by 2.5 LOD points and account for an additional 0.7% of the variation in resistance to *M. citrifolia* toxin (data not shown).

Based on the sample size and environmental and genetic variances of our cross, a QTL with effect size of 0.12, where 0 is sensitive and 1 is resistant, would be detectable with 80% power, and a QTL with effect size of 0.130 would be detected with 90% power in our *D. simulans^Nueva^* backcross. The effect sizes of the five QTL that we report exceed the 90% power threshold (Figure S2). The best-fit model explains 38.9% of the phenotypic difference between species (Table 1).

### Tests of QTL-III*_sim_*a and the effect of the X chromosome

The location of our largest-effect QTL in the *D. simulans ^Nueva^* backcross differs from the large-effect locus described for octanoic acid sensitivity in larvae (Jones 2001) and in adults (Jones 1998). We therefore performed additional checks of our approach. First, we compared the genotype of a sample of our MSG-genotyped flies at ∼3:46,689,564 with an indel marker at 3:46,617,828 using PCR. The PCR-indel genotypes of all 179 genotyped individuals matched the genotypes produced by MSG (data not shown). To confirm the existence of QTL-III*_sim_*a, we next crossed a *D. simulans* strain. marked by the *cutsy* mutation and by *claret* (*ca*) at ∼3:49,032,000 (Kimble and Church 1983), to *D. sechellia^D1A1C^* and backcrossed F1 females to the marked *D. simulans^cutsy^*^,^ *^ca^* parental strain. The genetic map position of *cutsy* is 3-74 in *D. simulans* (Coyne 1997), which is approximately ∼3:42,000,000. We compared the frequency of each recessive marker in 242 F1 backcross progeny that were viable in *M. citrifolia* fruit from L2 to adulthood with the frequency of each marker in 872 larvae that were not exposed to *M. citrifolia* fruit. We found that *cutsy^+^* (Σ^2^ = 20.6, *P* = 5.8 × 10^−6^) was most strongly associated with resistance to *M. citrifolia* toxin, followed by *ca^+^* (Σ^2^ =6.2, *P* = 0.01). We next used the molecular markers at 3:46,617,828 to test for linkage to an additional 89 resistant and 91 sensitive *D. simulans* backcross progeny. We found strong linkage to this region (*P* = 4 × 10^−5^, Fisher’s exact test; Σ^2^ = 27, *P* = 2.06 × 10^−7^, chi-squared test; Table 2). These data confirm that QTL-III*_sim_*a is located near 3:46,617,828 and suggest that QTL-III*_sim_*a may be closer to ∼3:42,300,000 than to ∼3:49,032,000, (Table 2). Finally, we asked if *M. citrifolia* resistance in our assay is linked to *ebony* (*e*), because Jones (2001) found that a QTL linked to *e* confers resistance to octanoic acid in a larval assay. The *e* gene is located at 3:28,656,573 in *D. simulans*. Like Jones (2001), we also find that *M. citrifolia* resistance is linked to *e* when we compare the frequency of *e* in resistant adults to the frequency of *e* in untreated adults. However the linkage to *e* is weaker than the linkage between resistance and *cutsy*, *ca*, or 3:46,617,828 (Table 3; Σ^2^ = 5.08, *P* = 0.02).

**Table 2:** Linkage of markers on 3R to *M. citrifolia* resistance

Location*^a^*	Marker*^b^*	Observed	Expected	Σ^2^	*P**^c^*
∼3:42,000,000	*cutsy^+^*	156	121	20.6	5.8 × 10^−6^
	*cutsy^−^*	86	121		
3:46,617,828	*indel^e^*	69	46	27.0	2.1 × 10^−7^
	*indel^i^*	20	44		
∼3:50,000,000	*ca^+^*	137	118	6.2	0.01
	*ca^−^*	105	124		

a Location in the *D. melanogaster* reference genome.

b For *cutsy* and *claret* (*ca*), inheritance of a second copy of the recessive marker from *D. simulans*. *Indel^e^* indicates inheritance of the *D. sechellia* variant of the indel polymorphism and *indel^i^* indicates the inheritance of a second copy of the *D. simulans* indel polymorphism.

c Chi-squared test.

**Table 3:** M. *citrifolia* linkage to *ebony (e)*

Location*^a^*	Marker*^b^*	Observed	Expected*^c^*	Σ^2^	*P**^d^*
∼3:28,656,573	*e^+^*	55	70	5.08	0.02
	*e^−^*	151	136		

a The location in the current *D. simulans* genome is: Scf_3R:4,244,000.

b Inheritance of a second copy of the recessive marker from *D. simulans*.

c Based on frequency of markers observed in 863 control larvae that were handled similarly but not exposed to *M. citrifolia* fruit.

d Chi-squared test.

Our analysis of both the *D. simulans* backcross and the *D. sechellia^w30^* backcross revealed resistance loci on the X chromosome, which contradicts previous work (Jones 2001). As an additional test of our QTL model, we compared the survivorship of male and female larvae from reciprocal F1 crosses in intermediate levels of octanoic acid. When the F1 cross is performed with a *D. sechellia* female and *D. simulans* male, both male and female offspring will inherit an X chromosome from *D. sechellia*. If, however, the cross is performed with a *D. sechellia* male and a *D. simulans* female, the female offspring will inherit an X chromosome from *D. sechellia*, whereas the male will inherit a Y chromosome from *D. sechellia*. We generated F1 offspring from crosses between inbred strains of *D. sechellia*, (D1A1C) and *D. simulans*, (A2A2B) and found that inheritance of a *D. sechellia* X chromosome greatly improved the survivorship of male larvae. At 0.4% octanoic acid dissolved in fly media, the number of males and females that survived treatment when the mother was *D. sechellia^D1A1C^* was similar (21 males and 15 females/96 larvae). However, a smaller proportion of males survived treatment when *D. simulans^A2A2B^* was the mother of F1 larvae (Table 4; *P* = 0.0002, Fisher’s exact test). We found similar results at 0.5% octanoic acid, although the overall survivorship was lower (Table 5; *P* = 0.01, Fisher’s exact test). We also found a similar pattern in which females had greater survivorship than males, with the offspring from the cross being female *D. simulans^Nueva^* × male *D. sechellia^w30^* in *M. citrifolia* (*P* = 9.36 × 10^−7^, Fisher’s exact test, N = 141 larvae in *M. citrifolia vs.* N = 371 control larvae). These data indicate that the X chromosome from *D. sechellia* has a significant effect on resistance to octanoic acid and *M. citrifolia* that is not strain-specific. Finally, the combined survivorship of both males and females in octanoic acid was not higher among F1 larvae from crosses with *D. sechellia* as the mother (Tables 4–5), suggesting that the reported maternal effect (Jones 2001) has dissipated by the second larval instar.

**Table 4:** Survivorship of larvae from reciprocal F1 crosses that were exposed to 0.4% octanoic acid

Cross*^a^*	Male *D. simulans^A2A2B^* × Female *D. sechellia^D1A1C^*	Male *D. sechellia^D1A1C^* × Female *D. simulans^A2A2B^*	*P**^b^*
Female	40	15	0.0002
Male	9	21	
Total larvae	100	96	

a Reciprocal crosses between *D. simulans^A2A2B^* and *D. sechellia^D1A1C^*.

b Fisher’s exact test.

**Table 5:** Survivorship of larvae from reciprocal F1 crosses that were exposed to 0.5% octanoic acid

Cross*^a^*	Male *D. simulans^A2A2B^* × Female *D. sechellia^D1A1C^*	Male *D. sechellia^D1A1C^* × Female *D. simulans^A2A2B^*	*P**^b^*
Female	19	15	0.01
Male	2	12	
Total larvae	100	186	

a Reciprocal crosses between *D. simulans^A2A2B^* and *D. sechellia^D1A1C^*.

b Fisher’s exact test.

## Discussion

We performed a high-resolution genetic analysis of the recently evolved resistance to *M. citrifolia* in *D. sechellia* larvae. Our work extends the pregenomic analysis of Jones (2001), which was performed with just a few visible markers and purified octanoic acid. By contrast, we use hundreds of thousands of genomic markers to identify loci that impart resistance to the toxic fruit. We find that resistance to *M. citrifolia* during the period from the second instar to puparium formation is composed of two major effect loci as well as multiple smaller-effect loci. For the *D. sechellia* backcross, two to three QTL contributed to resistance on the third chromosome (QTL-III*_sec_*a—c) as well as a region on the X chromosome (QTL-X*_sech_*; Figure 4). For the *D. simulans* backcross we identified two large-effect loci, one at ∼3:46,854,000 bp (QTL-III*_sim_*a; LOD = 59.2; Figure 5), another at ∼X:14,774,000 (QTL-Xsim; LOD = 20.2), and three regions of smaller effect that are located on 2L (QTL-II*_sim_*a), 2R (QTL-II*_sim_*b), and on 3L (QTL-III*_sim_*b; Figure 5). QTL-III*_sim_*a is within an interval that overlaps with QTL-III*_sec_*c, which may indicate that the two QTL are the same locus that acts additively (Table 6; Figure S3; Figure S4). Otherwise, C.I.s of the QTL from the two backcrosses do not overlap. We found no evidence for significant epistasis between any QTL. Additional checks of our locus of largest effect, QTL-III*_sim_*a, using visible and molecular markers confirm that the locus that confers resistance to *D. sechellia^w30^* is between ∼3:42,000,000 and 3:46,617,828, but closer to 3:46,617,828.

**Table 6:** Locations of QTL peaks and C.I. based on a 1.5 LOD drop from the peak value

QTL	Peak*^a^*	Range*^a^*	LOD*^b^*
QTL-X*_sec_*	∼X:10,290,000	∼X:4,975,000–11,153,000	6.5
QTL-III*_sec_*a	∼3L:15,680,000	∼3L:15,166,000–20,170,000	20.2
QTL-III*_sec_*b	∼3R:15,115,000	∼3R:14,951,000–15,324,000	17.0
QTL-III*_sec_*c	∼3R:19,027,000	∼3R:18,675,000–19,302,000	12.8
QTL-X*_sim_*	∼X:14,373,000	∼X:13,846,000–15,427,000	20.5
QTL-II*_sim_*a	∼2L:9,072,000	∼2L:8,438,000–12,131,000	6.3
QTL-II*_sim_*b	∼2R:19,461,000	∼2R:18,492,000–21,111,000	8.2
QTL-III*_sim_*a	∼3R:21,925,000	∼3R:21,461,000–22,067,000	59.2
QTL-III*_sim_*b	∼3L:5,075,000	∼3L:4,447,000–5,574,000	6.2

A list of genes that lie within this range for each QTL given in the supplementary information.

a Locations are derived from the *D. simulans* genome coordinates from Hu *et al.* (2012).

b LOD score from 1D QTL scan.

Although the set of loci that we detected overlap with the regions identified by Jones (2001), the two sets are not identical. For the *D. simulans* backcrosses, both studies find a major effect locus on 3R (Figure 5; Table 1), but Jones (2001) finds that the major effect locus is near *e*, and a region near QTL-III*_sim_*a was not significant. Another major difference between our QTL map and that of Jones (2001) is the effect of the X chromosome. We found significant QTL peaks on the X chromosome in both the *D. simulans* and *D. sechellia^w30^* backcrosses, whereas Jones (2001) did not detect any effects on the X chromosome (Figures 4–5). Like Jones (2001), we also found significant regions on 2R and 2L in the *D. simulans* backcross, although Jones found that the 2R region interacts with sex and with the QTL on 3R. In addition, Jones (2001) detected epistasis between 2L and 2R in the *D. sechellia* backcross, whereas we did not detect significant epistasis between any loci in either backcross. These differences may be attributable to the vastly different datasets used in the two experiments. The analyses of Jones (2001) were conducted with large numbers of recombinants; 14,339 *D. simulans* backcross larvae and 2252 *D. sechellia* backcross larvae. Therefore, his study would be expected to detect smaller effect loci and interactions. However, the study by Jones (2001), which used all the available visible markers for these species, consisted of just 11 markers in the *D. simulans* backcross and six markers in the *D. sechellia* backcross. Simulations that vary marker spacing show that although high marker density does not improve the power to detect loci, it has a significant effect on the precision of QTL localization (Darvasi *et al.* 1993; Stange *et al.* 2013). Hence, the Jones (2001) study is well-powered to detect small effect loci, but the locations of the QTL will be far less precise. Although our study is also well-powered, the extremely high marker density of our QTL analysis should predict QTL locations with much greater accuracy. We have therefore only compared QTL locations between the two studies in the broadest terms.

Several biological factors could also account for the discrepancies between our QTL map and the previous analysis by Jones (2001). First, we used fresh-frozen *M. citrifolia* for our assays, whereas Jones used purified octanoic acid in fly food. Although previous work (Legal *et al.* 1994) has established that octanoic acid is the toxic component of *M. citrifolia* fruit, other components of the fruit may contribute to the toxicity or uptake of octanoic acid. Synergy between two compounds, one which is toxic and a second that is not toxic on its own, is a well-established effect used in pest control (Bernard and Philogene 1993). Second, the two analyses may test different phases of development. Jones (2001) allowed females to oviposit on control or octanoic acid–containing medium and then recorded the visible markers of emerging adults. *D. simulans* embryos are highly sensitive to *M. citrifolia* (R’kha *et al.* 1991), although *D. sechellia* embryos are largely resistant. The lethality of octanoic acid decreases over time, presumably as the semivolatile compound dissipates. Therefore, in the Jones (2001) assay, embryonic resistance factors would be under the greatest selection whereas later stages of development would experience increasingly lower effective octanoic acid concentrations. Jones (2001) also provides strong evidence for a maternally inherited resistance factor, which further complicates genetic mapping. A locus that encodes a maternally provided factor would not appear in selection experiments if the offspring have resistant mothers. Therefore, any differences between the set of larval resistance factors described here and those described by Jones (2001) could be due to maternally provided factors, selection upon different stages of resistance, or any combination of the two. Finally, we compare the genotypes of resistant larvae with those of sensitive larvae for our QTL analyses, whereas Jones (2001) compares the genotypes of resistant larvae with those of larvae that have not been exposed to octanoic acid. Interestingly, linkage to the region containing *e* was not significant in our QTL map, but it was significant when we used untreated backcross progeny as a control (Figure 5;Table 3). One possible explanation for the differences in significance for a QTL near *e* could indicate that two different types of loci are detected depending on the group that is used as a basis for comparison. For instance, the QTL near *e* could be an enzyme that is used to detoxify octanoic acid. In this scenario, a *D. sechellia* enzyme would improve the viability of larvae, but having *D. simulans* copies would not increase the sensitivity to octanoic acid. However, the presence of a *D. simulans* target site in a receptor, for instance, could make the larva more sensitive to octanoic acid by providing a site for inappropriate stimulation or inactivation.

In contrast to larval resistance, the resistance to volatile octanoic acid in adults is conferred through dominant loci on the second, third, and X chromosomes (Jones 1998). The resistance factor on chromosome 2 had too small of an effect to be mapped, but the loci on X and III were further resolved with visible markers (Jones 1998). A region on the right arm of chromosome 3 that is linked to *e* had the greatest effect, and recent work has fine-mapped this locus to the interval bounded by ∼3:26,136,000 and ∼3:26,315,000 (Hungate *et al.* 2013). Interestingly, our *D. simulans* backcross QTL map for larval resistance overlaps with a locus on the X chromosome discovered by Jones (1998) for adult resistance: a region between *miniature* (∼X:11,700,000) and *forked* (∼X:17,130,000). We find a broad significant region from ∼X:10,000,000 to ∼X:18,200,000 that peaks at ∼X:14,358,268. The lack of agreement between the set of loci uncovered for larval and adult resistance suggests a stepwise path toward adaptation to the toxic fruit by *D. sechellia*. Rather than a single, large-effect locus that would confer resistance to *M. citrifolia* toxin at each stage, the *D. sechellia* genome appears to have invented multiple, stage-specific resistance mechanisms. One such mechanism has been suggested for embryonic resistance. *D. sechellia* females are ovoviviparous and hold fertilized eggs until the later stages of embryogenesis, when the embryonic epidermis secretes the first instar cuticle (Markow *et al.* 2009; Lavista-Llanos *et al.* 2015) . Our data with *D. simulans* show that larvae are most sensitive to octanoic acid during molts. Apparently, the embryonic cuticle, like the larval cuticle, protects *Drosophila* from the toxic effects of octanoic acid.

The response of insect populations to insecticide treatment may provide insight into how resistance to *M. citrifolia* toxin may have evolved in *D. sechellia*. Exposures to insecticide concentrations that lie within the distribution of viability tend to produce resistance that is based on multiple loci, each of small effect (McKenzie *et al.* 1992). Such variation has been found to regulate the expression level or copy number of detoxifying enzymes, such as the cytochrome P450s, and by other metabolic enzymes, such as carboxylases and esterases (McKenzie and Batterham 1994; Ranson *et al.* 2002; Ffrench-Constant *et al.* 2004). Selection outside of the viability distribution with very high levels of insecticide, however, tends to produce large-effect loci conferred by amino acid substitutions of single genes that are the targets of pesticides. These targets include ligand-gated ion channels, like the GABA receptor subunit in which an amino acid replacement confers resistance to dieldrin (Ffrench-Constant and Roush 1991), or a voltage-gated sodium channel in which resistance to DDT is conferred by either of two amino acid replacements (Williamson *et al.* 1993; Miyazaki *et al.* 1996) . The genetic architecture of resistance to *M. citrifolia* fruit toxin in *D. sechellia* that we describe here bears hallmarks of both types of selection; one large-effect locus on 3R accounts for 24% of the phenotypic difference between *D. simulans^Nueva^* and *D. sechellia^w30^*, but in the best-fit model additional smaller-effect loci also confer resistance (Table 1).

Data from selection experiments suggest that complete resistance did not arise only through consolidation of existing variation in resistance alleles alone. Colson (2004) selected for increased resistance in a cosmopolitan strain of *D. simulans* for 20 generations and found that resistance to octanoic acid rapidly increased by 18% before it plateaued at a fraction of the resistance seen in *D. sechellia*. These data show that either the variation within the cosmopolitan strain that was used is not representative of the variation within the common *D. simulans*–*D. sechellia* ancestor or the resistance in *D. sechellia* arose through new mutation(s). Interestingly, two regions discovered in these selection experiments overlap substantially with QTL-II*_sim_* at cytological location 57C and with QTL-X*_sim_* at cytological location 13D (Colson 2004). Colocalization of our small-effect loci with the evolved resistance regions of Colson (2004) supports a scenario in which the smaller-effect loci discovered in our experiments are the products of selection for octanoic acid resistance within the viability distribution of *D. simulans*.

The genetic complexity of resistance to *M. citrifolia* fruit in *D. sechellia* appears to be a barrier to full introgression of this trait into *D. simulans*. Amlou *et al.* (1997) tried to introgress octanoic acid resistance into *D. simulans* by backcrossing resistant adult hybrids to *D. simulans*. Despite strong directional selection, resistance decreased with each generation to *D. simulans* levels. Our own efforts to introgress the resistance phenotype into *D. simulans^Nueva^* (phenotype-based introgression) did not progress beyond one generation of selection upon ripe *M. citrifolia* (data not shown). However, the lack of epistasis between the five major QTL in our *D. simulans* backcross suggests that partial resistance could easily be introgressed from *D. sechellia* to *D. simulans*. Partial resistance would improve the viability of hybrid flies on fermenting fruit, which has been shown to have lower levels of octanoic acid (Legal *et al.* 1994, 1999; Farine *et al.* 1996). Such introgression of partial resistance may occur routinely in the Seychelles, where *D. simulans* and *D. sechellia* coexist. Matute and Ayroles (2014) recently showed that *D. simulans* and *D. sechellia* hybrids are prevalent in some of the islands in the Seychelles. They also found that F1 *D. simulans*/*D. sechellia* hybrids and morphologically *D. simulans* flies are found on *M. citrifolia* fruit, although the stage of fruit maturation was not reported (Matute and Ayroles 2014).

Previous studies of octanoic acid resistance in *D. sechellia* have identified other major effect loci, but none that overlaps with QTL-III*_sim_*a. The Jones (2001) analysis of larval resistance identified a locus on 3R near *Ubx* (∼3R:8,740,000), but this gene is ∼12.7 Mbp from the C.I. from QTL-III*_sim_*a. A significant region was also discovered on 3R that conveys resistance to volatile octanoic acid in adults (Jones 1998; Hungate *et al.* 2013), although this region, which is located near the centromere between ∼3R:1,900,000 and 2,080,000 is ∼18 Mbp from QTL-III*_sim_*a. Studies of the *Indifferent* (*Indf*) locus in *D. melanogaster* uncovered resistance to octanoic acid that is comparable to the level of resistance seen for *D. sechellia* (Legal *et al.* 1999). *Indf* has been localized to 96A2-7, a 143-kb region that is within 1 Mbp of the C.I. of QTL-III*_sim_*a.

QTL analyses typically produce C.I.s that are too large to implicate candidate genes. In the case of QTL-III*_sim_*a, the C.I. created by a 1.5 LOD score drop from the peak value of the locus spans from ∼3:46, 416,000 to 47,021,000, ∼0.61 Mbp. This interval contains 50 protein-coding genes, only 23 of which are named (File S4, File S5, File S6, File S7, File S8, File S9, File S10, File S11). Of these, three genes are potential toxin targets that are characteristic of exposure to very high levels of pesticides (McKenzie and Batterham 1994). The set includes a G-protein-coupled receptor that has an unknown function and *pickpocket15* (*ppk15*), a degenerin/epithelial sodium channel. Although a ligand for Ppk15 has not been identified, other Ppk receptors are used to sense water (Cameron *et al.* 2010) or to respond to long chain fatty acids during courtship and larval aggregation (Mast *et al.* 2014; Thistle *et al.* 2012; Toda *et al.* 2012). Although many genes within the 1.5 LOD score drop C.I. were metabolic enzymes, none was a carboxylase or esterase or member of the cytochrome P450 superfamily. In addition to the unnamed protein coding genes there are also eight nonprotein coding genes. Additional work with introgression lines or *D. melanogaster* deficiency strains will be needed to further resolve this interval with fine scale mapping. These experiments are currently underway in our laboratory.

## 

## References

[bib1] Amlou M. Pla E. Moreteau B. David J. R., 1997 Genetic analysis by interspecific crosses of the tolerance of *Drosophila sechellia* to major aliphatic acids of its host plant. Genet. Sel. Evol. 29: 511–522.

[bib2] Andolfatto P. Davison D. Erezyilmaz D. Hu T. Mast J., 2011 Multiplexed shotgun genotyping for rapid and efficient genetic mapping. Genome Res. 21: 610–617. 21233398 10.1101/gr.115402.110 PMC3065708

[bib3] Baird N. A., P. D., Etter, T. S. Atwood, M. C. Currey., A. L. Shiver, Z. A. Lewis, E. U. Selker, W. A. Cresko and E. A. Johnson, 2008 Rapid SNP discovery and genetic mapping using sequenced RAD markers. PLoS One 10.1371/journal.pone.0003376. PMC2557064 18852878

[bib4] Bernard C. B. Philogene B. J., 1993 Insecticide synergists: role, importance, and perspectives. J. Toxicol. Environ. Health 38: 199–223. 8433403 10.1080/15287399309531712

[bib5] Broman K. W. Speed T. P., 1999 A review of methods for identifying QTLs in experimental crosses. Stat. Mol. Biol. 33: 114–141.

[bib6] Broman K. Wu H. Sen S. Churchill G., 2003 R/QTL mapping in experimental crosses. Bioinformatics 19: 889–890. 12724300 10.1093/bioinformatics/btg112

[bib7] Cameron P. Hiroi M. Ngai J. Scott K., 2010 The molecular basis for water taste in Drosophila. Nature 465: 91–95. 20364123 10.1038/nature09011 PMC2865571

[bib8] Cande J. Andolfatto P. Prud’homme B. Stern D. L. Gompel N., 2012 Evolution of multiple additive loci caused divergence between Drosophila yakuba and D. santomea in wing rowing during male courtship. PLoS One 7: e43888 10.1371/journal.pone.0043888. 22952802 PMC3431401

[bib9] Chan-Blanco Y. Vaillant F. Perez A. M. Reynes M. Brillouet J., 2006 The noni fruit (Morinda citrifolia L.): A review of agricultural research, nutritional and therapudic properties. J. Food Compos. Anal. 19: 645–654.

[bib10] Chouard T., 2010 Evolution: Revenge of the hopeful monster. Naure 463: 864–867. 10.1038/463864a 20164895

[bib11] Colson I., 2004 Drosophila simulans’ response to laboratory selection for tolerance to a toxic food source used by its sister species D. sechellia. Evol. Ecol. 18: 15–28.

[bib12] Coyne, J. 1997 1.2 Flybase Stock List. Available at http://flybase.org/reports/FBrf0098117.html. Accessed: August 10, 2015.

[bib13] Darvasi A. Weinreb A. Minke V. Weller J. I. Soller M., 1993 Detecting marker-QTL linkage and estimating QTL gene effect and map location using a saturated genetic map. Genetics 134: 943–951. 8349116 10.1093/genetics/134.3.943 PMC1205528

[bib14] Erezyilmaz D. F. Stern D. L., 2013 Pupariation site preference within and between Drosophila sibling species. Evolution 67: 2715–2727. 10.1111/evo.12146 24033178

[bib15] Farine J. Legal L. Moretaeau B. Le Quere J., 1996 Volatile components of ripe fruits of *Morinda citrifolia* and their effects on *Drosophila*. Phytochemistry 41: 433–438.

[bib16] Ffrench-Constant R. H. Roush R. T., 1991 Gene mapping and cross-resistance in cyclodiene insecticide-resistant Drosophila melanogaster (Mg). Genet. Res. 57: 17–21. 1904046 10.1017/s0016672300028986

[bib17] Ffrench-Constant, R, P. J. Daborn, and G. Le Goff. 2004 The genetics and genomics of insecticide resistance. Trends Genet. 20:163–170. 10.1016/j.tig.2004.01.003 15036810

[bib18] Forister M. L. Dyer L. A. Singer M. S. Stireman J. O. III Lill J. T., 2012 Revisiting the evolution of ecological specialization, with emphasis on insect-plant interactions. Ecology 93: 981–991. 22764485 10.1890/11-0650.1

[bib19] Garrigan D. Kingan S. B. Geneva A. J. Andolfatto P. A. Clark A. G., 2012 Genome sequencing reveals complex speciation in the Drosophila simulans clade. Genome Res. 22: 1499–1511. 22534282 10.1101/gr.130922.111 PMC3409263

[bib20] Higa I. Fuyama Y., 1993 Genetics of food preferences in *Drosophila sechellia*. I. Responses to food attractants. Genetica 88: 129–136. 8224853 10.1007/BF02424469

[bib21] Hu T. T. Eisen M. B. Thornton K. R. Andolfatto P. A., 2012 A second generation assembly of the Drosophila simulans genome provides new insights into patterns of lineage-specific divergence. Genome Res. 23: 89–98. 22936249 10.1101/gr.141689.112 PMC3530686

[bib22] Hungate E. A. Earley E. J. Boussy I. A. Turissini D. A. Ting C. T., 2013 A locus in Drosophila sechellia affecting tolerance of a host plant toxin. Genetics 195: 1063–1075. 24037270 10.1534/genetics.113.154773 PMC3813837

[bib23] Jones C. D., 1998 The genetic basis for *Drosophila sechellia’s* resistance to a host plant toxin. Genetics 149: 1899–1908. 9691045 10.1093/genetics/149.4.1899 PMC1460277

[bib24] Jones C. D., 2001 The genetic basis of larval resistance to a host plant toxin in Drosophila sechellia. Genet. Res. 78: 225–233. 11865712 10.1017/s0016672301005298

[bib25] Kimble M. Church K., 1983 Meiosis and early cleavage in Drosophila melanogaster eggs: effects of the claret-non-disjunctional mutation. J. Cell Sci. 62: 301–318. 6413518 10.1242/jcs.62.1.301

[bib26] Kliman R. M. Andolfatto P. Coyne J. A. Depaulis F. Kreitman M., 2000 The population genetics and the origin and divergence of the Drosophila simulans complex of species. Genetics 156: 1913–1931. 11102384 10.1093/genetics/156.4.1913 PMC1461354

[bib27] Lachaise D. David J. R. Lemeunier F. Tsacas L., 1986 The reproductive relationships of *Drosophila sechellia* with *D. mauritiana*, *D. simulans*, and *D. melanogaster* from the Afrotropical region. Evolution 40: 262–271. 10.1111/j.1558-5646.1986.tb00468.x 28556049

[bib48] Lavista-Llanos S. Svatos A. Kai M. Riemenperger T. Birman S., 2014 Dopamine drives Drosophila sechellia adaptation to its toxic host eLife DOI: 10.766/eLife.03785 10.7554/eLife.03785 PMC4270095 25487989

[bib28] Legal L. David J. R. Jallon J. M., 1992 Toxicity and attraction effects produced by *Morinda citrifolia* fruits on the *Drosophila melanogaster* complex of species. Chemoecology 3: 125–129.

[bib29] Legal L. Chappe B. Jallon J., 1994 Molecular Basis of *Morinda citrifolia* (L.): Toxicity on *Drosophila*. J. Chem. Ecol. 20: 1931–1943. 24242720 10.1007/BF02066234

[bib30] Legal L. Moulin B. Jallon J. M., 1999 The relation between structures and toxicity of oxygenated aliphatic compounds homologous to the insecticide octanoic acid and the chemotaxis of two species of *Drosophila*. Pestic. Biochem. Physiol. 65: 90–101.

[bib32] Louis J. David J. R., 1986 Ecological specialization in the *D. melanogaster* species subgroup: A case study of *D. sechellia*. Acta Oecol. Gen. 7: 215–229.

[bib47] Markow T. A. Beall. S. Matzkin L. M. 2009 Egg size, embryonic development time and ovoviviparity in *Drosophila* species. J. Evol. Biol. 22:430–434. 19032497 10.1111/j.1420-9101.2008.01649.x PMC13729255

[bib33] Mast J. D. De Moraes C. M. Albom H. T. Lavis L. D. Stern D. L., 2014 Evolved differences in larval social behavior mediated by novel pheromones. eLife. 10.7554/eLife.04205. PMC4270068 25497433

[bib34] Matute D. R. Ayroles J. F., 2014 Hybridization occurs between *Drosophila simulans* and *D. sechellia* in the Seychelles archipelago. J. Evol. Biol. 27: 1057–1068. 24773151 10.1111/jeb.12391

[bib35] McKenzie J. A. Batterham P., 1994 The genetic, molecular and phenotypic consequences of selection for insecticide resistance. Trends Ecol. Evol. 9: 166–169. 21236810 10.1016/0169-5347(94)90079-5

[bib36] McKenzie J. A. Parker A. G. Yen J. L., 1992 Polygenic and single gene responses to selection for resistance to diazinon in *Lucilia cuprina*. Genetics 130: 613–620. 1551581 10.1093/genetics/130.3.613 PMC1204877

[bib37] Miyazaki M. Ohyama K. Dunlap D. Y. Matsumura F., 1996 Cloning and sequencing of the para-type sodium channel gene from susceptible and *kdr*-resistant German cockroaches (*Blattella germanica*) and house fly (*Musca domestica)*. Mol. Gen. Genet. 252: 61–68. 8804404

[bib38] R’Kha, S., P. Capy, and J. David., 1991 Host-plant specialization in the Drosophila melanogaster species complex: A physiological, behavioral, and genetical analysis. Proc. Natl. Acad. Sci. USA 88: 1835–1839. 1900368 10.1073/pnas.88.5.1835 PMC51120

[bib39] Ranson H. Claudianos C. Ortelli F. Abgrall C. Hemingway J., 2002 Evolution of supergene families associated with insecticide resistance. Science 298: 179–181. 12364796 10.1126/science.1076781

[bib40] Sen S. Satagopan J. M. Broman K. W. Churchill G. A., 2007 R/qtlDesign: inbred line cross experimental design. Mamm. Genome 18: 87–93. 17347894 10.1007/s00335-006-0090-y PMC2366108

[bib41] Smith R. Sheppard K. DiPetrillo K. Churchill G., 2009 Quantitative trait locus analysis using J/qtl. Methods Mol. Biol. 573: 175–188. 19763928 10.1007/978-1-60761-247-6_10

[bib42] Stange M. Utz H. F. Schrag T. A. Melchinger A. E. Wurschum T., 2013 High-density genotyping: an overkill for QTL mapping? Lessons learned from a case study in maize and simulations. Theor. Appl. Genet. 126: 2563–2574. 23860723 10.1007/s00122-013-2155-0

[bib43] Thistle R. Cameron P. Ghorayashi A. Dennison L. Scott K., 2012 Contact chemoreceptors mediate male-male repulsion and male-female attraction during courtship. Cell 149: 1140–1151. 22632976 10.1016/j.cell.2012.03.045 PMC3365544

[bib44] Toda H. Zhao X. Dickson B. J., 2012 The Drosophila female aphrodisiac pheromone activates ppk23(+) sensory neurons to elicit male courtship behavior. Cell Reports 1: 599–607. 22813735 10.1016/j.celrep.2012.05.007

[bib45] Tsacas L. Bachli G., 1981 D. sechellia n. sp huitieme espece du sous-groupe melanogaster des iles Seychelles (Diptera Drosophilidae). Rev. Fr. Entomol. 3: 146–150.

[bib46] Williamson M. S. Denholm I. Bell C. A. Devonshire A. L., 1993 Knockdown resistance (kdr) to DDT and pyrethroid insecticides maps to a sodium channel gene locus in the housefly (*Musca domestica*). Mol. Gen. Genet. 240: 17–22. 8101963 10.1007/BF00276878

